# Veterinarians' perspective on telemedicine in Germany

**DOI:** 10.3389/fvets.2023.1062046

**Published:** 2023-02-15

**Authors:** Björn Becker, Andrea Tipold, Jan Ehlers, Christin Kleinsorgen

**Affiliations:** ^1^Centre for E-Learning, Didactics and Educational Research, University of Veterinary Medicine, Hannover, Germany; ^2^Veterinary Practice B. Becker, Heek, Germany; ^3^Clinic for Small Animals, University of Veterinary Medicine, Hannover, Germany; ^4^Department of Medical, Faculty of Health, Didactics and Educational Research in Health Care, Witten/Herdecke University, Witten, Germany

**Keywords:** telemedicine, veterinary telemedicine, teleconsultation, teleradiology, digitalization

## Abstract

**Introduction:**

Information on the use of telemedical approaches in the context of veterinary medicine is evolving. As in human medicine, veterinary medicine is subject to an increasing digitalization effort. The aim of the current study was to investigate the perspective of German veterinarians regarding their awareness and usage of telemedical approaches. Furthermore, the degree of implementation of different digital approaches in the context of German veterinary medicine was elaborated.

**Methods:**

A literature review, that also aimed to address the necessary framework or standardization of these digitalization efforts and potential barriers such as legal or infrastructural aspects, provided information for the empirical research. Using a quantitative research approach, the perspective of German veterinarians was surveyed.

**Results:**

In total, responses from 169 veterinarians were analyzed. The results show that digital approaches were used by veterinarians and the usage was enhanced by the COVID-19 crisis.

**Discussion:**

However, the lack of a clear legal framework may be a significant barrier for further implementation. This survey provides a basis for a critical discussion on the use of veterinary telemedicine in Germany. The results may contribute to future strategies for the implementation and development of necessary policies, training, and service applications within Germany, which may be transferable for the profession in other countries.

## 1. Introduction

The German Medical Association presents a basic definition of telemedicine: “*Telemedicine is a collective term for various medical care concepts that have in common the principle approach of providing medical services for the health care of the population in the areas of diagnostics, therapy and rehabilitation as well as medical decision-making advice over spatial distances. Information and communication technologies are used for this purpose.”* (1). This means, for example, that a follow-up call after surgery also falls under this definition of telemedicine.

Telemedicine may be important in the future for everyday work in veterinary medicine (2). It offers various advantages for veterinarians, the patients and their owners, as patient information can be passed from one specialist to another much more easily, allowing faster or possibly more accurate diagnosis and more efficient treatment. Ideally, telemedicine can fill in gaps and provide better care and treatment for patients without owners or veterinarians having to make a time-consuming and often arduous journey to the city or far into the countryside. Therefore, telemedicine and the underlying concept of bringing information digitally to where it is needed are among the most groundbreaking developments in the field of medicine (3). Veterinary telemedicine can be a success factor by helping to reduce error rates, perform more procedures and improve the overall client-veterinarian relationship when applied appropriately (3). As an example, the authors Papageorges and Tilley cite the sharing of “radiograph, sonogram, and cytology to radiologists, cardiologists, and pathologists,” while other activities are carried out. Discussion with or reports by board-certified specialists allow for more comprehensive advice, diagnosis and a treatment plan even in remote areas (3, 25). However, examples listed are mainly individual and subjective opinions without statistical evidence or empirical research have been described. Video visits, remote patient monitoring and telecare are methods that have already found their way into human medicine and will increasingly influence veterinary medicine in years to come (2). Some veterinarians already offer their clients the possibility of tele-consultation, and presumably clients' demands will enhance the importance of telemedicine in the field of veterinary medicine in the medium term (2), while teleradiology is already established in daily work and companies offering teleradiology services exist (3–5). Teleradiological services include primary interpretation of images, systems to directly or indirectly send data to specialists, on-call coverage or second opinion consultations, as well as image storage and archival or database banking (5). Increasing pressure for innovations in the field of digital technologies and telemedicine are obvious (6–8). Smartphones, apps and camera systems are constantly improving and systems are being adapted and implemented also within veterinary medicine. IHS Technology suggested in their report that the number of patients using veterinary telemedicine services globally would grow from 350,000 in 2013 to 7 million in 2018 (7, 8). In the field of animal health, the innovation debate primarily revolves around veterinarians themselves who are considered to be innovation-friendly and open (6). Telemedicine has been described as one key development in 2018 by the Royal College of Veterinary Surgeons (RVCS) survey, which included more than one thousand participating British veterinarians. In that survey, a large proportion of participants rated telemedicine as a topic worth discussing, but concerns were also expressed (9). Those concerns about risks included “risk of error due to incomplete information, a lack of a physical examination, limitations to technology and issues relating to owner trust” (9). The survey also revealed uncertainty about the extent to which telemedicine services can and should be included in the Code to Professional Conduct for vets. However, some veterinary medicines (such as antiparasiticides) that could be prescribed remotely were endorsed.

Thus, the development of veterinary medicine seems to be in line with developments in human medicine where the widespread use of telemedicine approaches has been discussed with greater detail (10). Telemedicine and telehealth as a means of caring for hard-to-reach patients are attractive in many ways (10). These approaches can save time and money and reduce the inconvenience of travel for healthcare professionals as well as patients and clients. They can also make rare expertise more widely available by enabling better networking among colleagues and greater international availability of expert knowledge (10). In addition, telemedicine approaches can also help patients who would otherwise go untreated (10). At the same time, however, these developments are met with a high degree of mistrust, which seems to be based on several uncertainties, for example, regarding the issue of responsibility or cost factors (10). Due to lack of healthcare infrastructures and an increased risk of zoonotic diseases, telecommunication technologies, such as smartphones and digital cameras, are particularly useful in developing countries (11). Telehealth equipment can support early diagnosis, surveillance, warning or reporting and the prevention of zoonotic diseases, thereby playing an important role in One Health approaches (11).

The only exception here is teleradiology, which is an established branch in the field of telemedicine. The possibilities for increased productivity and expansion of diagnostic options due to digitalization, particularly in diagnostic imaging, were described as a pioneering future trend as early as 2001 (12). Numerous successful business models have been developed in the field of teleradiology, also in veterinary medicine. Corresponding teleradiology solutions also play an important role with regard to the increasing desire for teleworking and telecommuting in the context of the COVID-19 crisis (13, 14).

Veterinary medicine and animal husbandry are described as highly specialized fields encompassing the management and health care of various animal species: “disease diagnosis, treatment, and prevention; quality assessment of meat and food, including milk and dairy products; quarantine procedures; animal welfare; feed formulation and testing; technology dissemination; and teaching, training, innovation, generation, and transfer of knowledge or technologies for end users as well as administrators” (15). Accordingly, the critical role of veterinarians in meeting these growing responsibilities is complex. Despite this complexity, however, telemedicine, with the exception of teleradiology, still seems to be comparatively underutilized and, above all, insufficiently researched in the field of veterinary medicine (15).

Telemedicine seems to be rapidly gaining relevance, especially in the context of the COVID-19 crisis (16). As in other areas of social and economic life, the pandemic and the measures taken by governments led to increased demand and support for digitalization efforts (17). While home office or telework/telecommuting models increased in other sectors, there was also a trend in medicine to minimize avoidable physical interactions (18, 19). More fundamentally, many governments did not adequately explain the critical role of veterinary medicine. While medical staff in the human health care sector were clearly defined as key personnel who were accordingly exempt from lockdown measures, this was only partially clarified in veterinary medicine and related fields (20). Overall, telemedicine appears to be an emerging subfield in veterinary medicine as well, despite or even as a result of the COVID-19 pandemic (21).

While telemedicine in human medicine is already considered comparatively well researched, there seems to be a research gap in the field of veterinary medicine (15, 22). This lack of research in the field of veterinary telemedicine has already been criticized (23). Mars and Auer (2006) pointed out that telemedicine approaches have been used in veterinary medicine for a long time but are rarely discussed and researched. Modern telemedicine should be evaluated in detail and the billing of animal owners should be better clarified (23). In addition to the fundamental research gap on the question of the extent to which telemedicine has also found its way into the field of veterinary medicine and which approaches are used in practice in this context, the topic of the effects of the COVID-19 crisis also emerges: society as a whole seems to have experienced a digitalization surge in the last 2 years, which has also led to an increased use of remote communication, for example in the fields of medicine, education or business (24, 25).

In the context of this study, the extent to which veterinary medicine itself has been exposed to this digitalization push is elaborated, as the study was carried out during the pandemic as were the corresponding countermeasures. Therefore, the aim of this study was to investigate German veterinarians' perception of digital technologies and potential change in usage with regard to infrastructural, technical and legal frameworks using an online survey. Furthermore, it was investigated whether the attitude to telemedicine has an influence on the use of telemedicine and whether the COVID-19 pandemic has served as a catalyst for a change in awareness. Results shall inform about the developing of or improvement to guidelines and regulations to introduce and implement telemedical approaches in a more standardized manner in veterinary medical practices.

## 2. Method

An online survey (LimeSurvey^®^) was created on the basis of existing instruments such as the “Telehealth Practice Survey” (26), “a survey of knowledge and use of telehealth among veterinarians” (27) or the survey “Digitalisierungsreport” [“digitalization report,” (28)] to evaluate the attitude toward and the implementation of telemedical services among individuals in veterinary practice. Before publishing the current survey, it was pre-validated and piloted by expert staff from the University of Veterinary Medicine in Hannover as well as veterinary colleagues as potential respondents (29).

The survey consisted of four question groups:

1. Sociodemographic questions and general questions about the participating veterinary practice. Questions about age, gender, type and localization of the practice (rural or urban environment) and role in the clinic/practice are supplemented by questions about mainly treated species.
2. The perception and basic attitudes of the participating practitioners regarding digitalization in relation to the veterinary professional ethos were assessed using rating questions with a rating scale from “1 = strongly agree” to “4 = strongly disagree.”

Fifteen questions evaluated communication-related aspects. The participants rated communications tools using a scale that ranged from “1-mostly used” to “5-not specified.” The questions were separated into two main parts. The focus of the first parts was the communication management of a veterinarian in daily work life. The options for “communication” were “telephone,” “physically (which means “in the practice”),” “video chat,” “E-mail,” “messenger” (like WhatsApp, Facebook, SMS), “fax” and “letter.” In the second part it was asked how communication took place with new and existing customers.

3. General questions about the status of information and wishes for a legal framework regarding telemedicine in veterinary practices. With the aim to draw conclusions as to whether knowledge of the legal framework is a limiting or motivating factor for the use of telemedical solutions. To verify a possible uncertainty more precisely, the question was asked whether the participant believes that telemedicine is already sufficiently regulated in the professional code of conduct. The last question in this section dealt with suggestions from veterinarians regarding education and information from professional organizations.
4. COVID-19-related questions including questions about the influence of COVID-19 as a catalyst regarding the awareness and the use of telemedicine.

The survey was sent to veterinarian practitioners *via* social media in veterinarian expert groups (e.g., closed veterinary Facebook groups) and *via* newsletters by the local Veterinary Chamber of Lower Saxony between May 2020 and August 2020. Participants were asked to respond as honestly and applicably as possible based on their own experiences. It was pointed out that personal data would be collected, stored and analyzed respecting the General Data Protection Regulations (GDPR) and anonymity of all respondents. Results were evaluated anonymously and participants were free to discontinue participation in the survey at any time, which would result in the deletion of the data collected up to that point. All questions were asked only optionally, which meant that questions could be skipped and no answer was forced in order to continue processing the questionnaire. In addition, the answer option “no answer” was offered for each question to allow for abstention.

Raw data from the survey were first transferred to Microsoft Excel^®^ (2010 Microsoft Corporation). Sorting algorithms were used and questionnaires that were not completed to the end were filtered out. These were deleted and, based on the convenience sample, a descriptive analysis of the results was carried out and presented in tabular and graphical forms using Microsoft Excel^®^ (2010 Microsoft Corporation).

This study was conducted in accordance with the ethical standards of the University of Veterinary Medicine Hannover, Foundation. The Doctoral Thesis Committee of the university validated the project in accordance with the ethical guidelines regarding research with human participants and approved the study. The university's Data Protection Officer approved the project. The voluntarily participating persons consented to the processing of their data in accordance with the EU General Data Protection Regulation of 2018 (General Data Protection Regulation Art. 6 I 1 lit. e i.V.m. 89 and Lower Saxony Data Protection Act § 3 I 1 No. 1 NHG, § 13). Data processing was performed anonymously in accordance with the Data Protection Regulation of the university.

## 3. Results

### 3.1. Socio-demographic data

**Figure 1:**
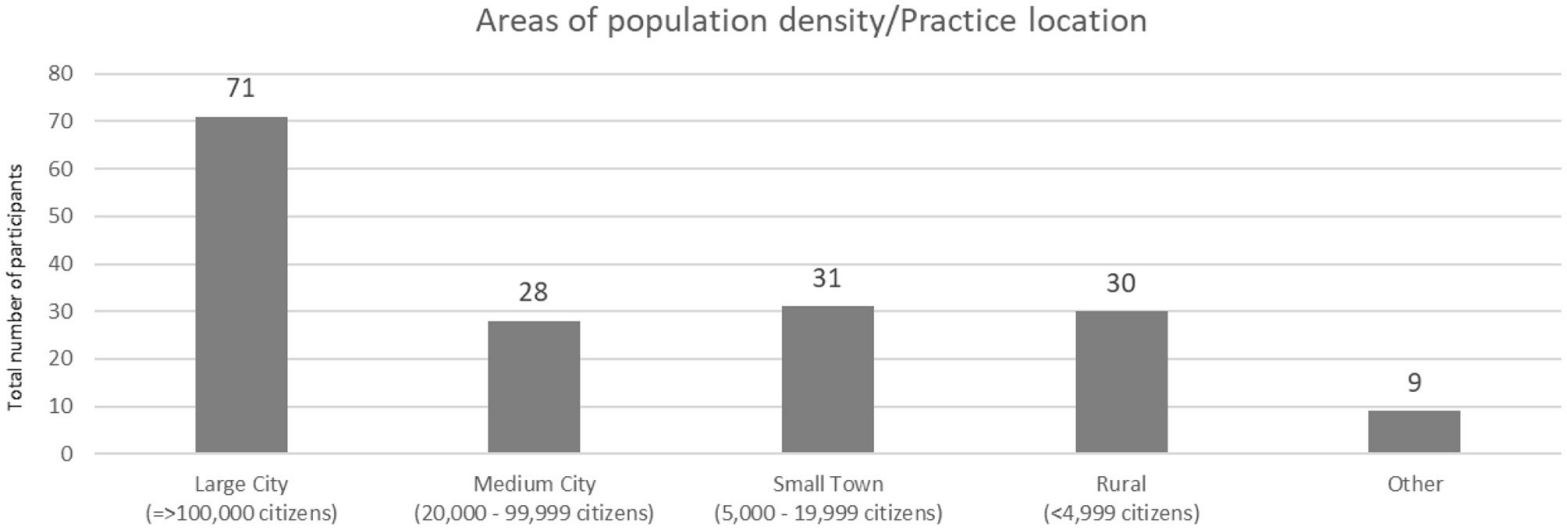
Distribution of respondents in different areas of population density (*N* = 169).

A total of 300 veterinarians started to answer the survey. However, only 169 respondents completed the entire survey and were included in further analyses. The gender ratio within the sample was unbalanced, with 114 females (67.5 %) and 55 males (32.5 %). Participating veterinarians were on average 45.19 years old (SD = 11.96 years). Figure 1 shows the distribution of respondents in different areas of population density.

A large proportion of respondents reported working in a large city, while the rest were distributed among medium-sized cities, small towns and rural areas (Figure 1).

**Figure 2:**
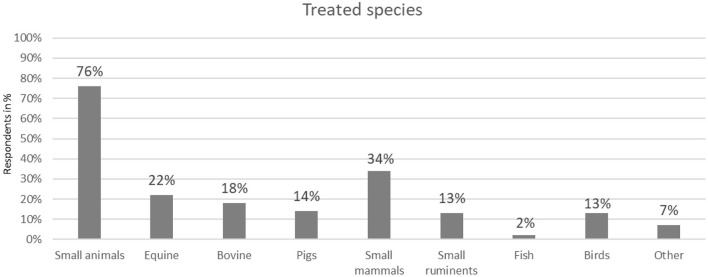
Animals treated in practices by respondents (*N* = 169, multiple answers possible).

Figure 2 shows that the majority of participating veterinary practices treated small animals (76 %), but participants also came from the livestock (45 %), equine (22 %) and small pet sectors (34 %). Specialty practices (fish and birds) were also among the participants (15 %).

**Figure 3:**
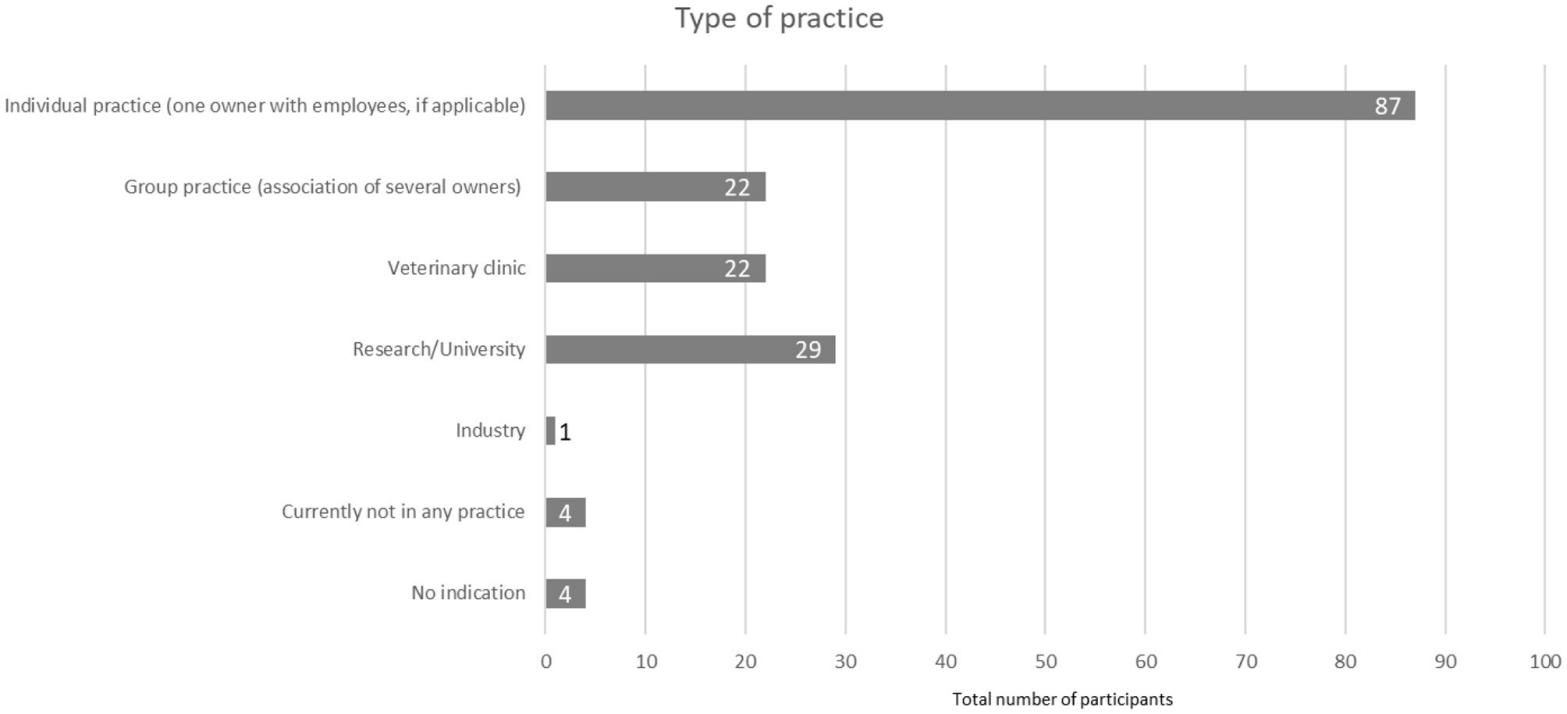
Type of practice in which respondents worked (*N* = 169).

The majority of participants worked in individual practices (*n* = 87, 51.5 %), followed by university (*n* = 29, 17.2 %), group practices (*n* = 22, 13 %) and clinics (*n* = 22, 13 %) and a small number did not work in any practice (*n* = 4, 2.4 %) (Figure 3).

**Figure 4:**
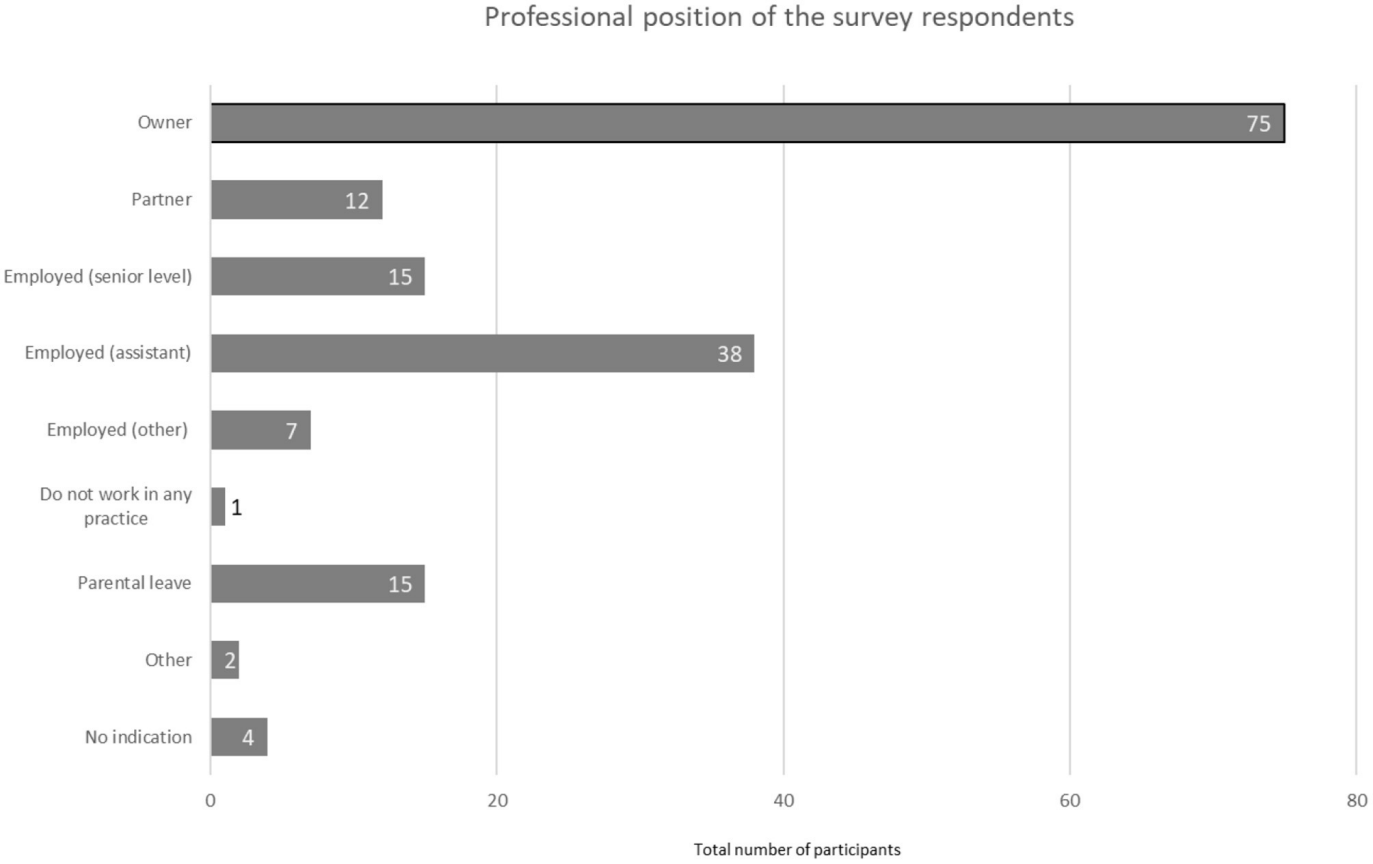
Professional position of the survey respondents (*N* = 169).

The majority of respondents were practice owners (*n* = 75, 44.4 %), followed by employed physicians (*n* = 38, 22.5 %) and those on parental leave (*n* = 15, 8.9 %) (Figure 4).

### 3.2. Questions about communication with the animal owners

**Figure 5:**
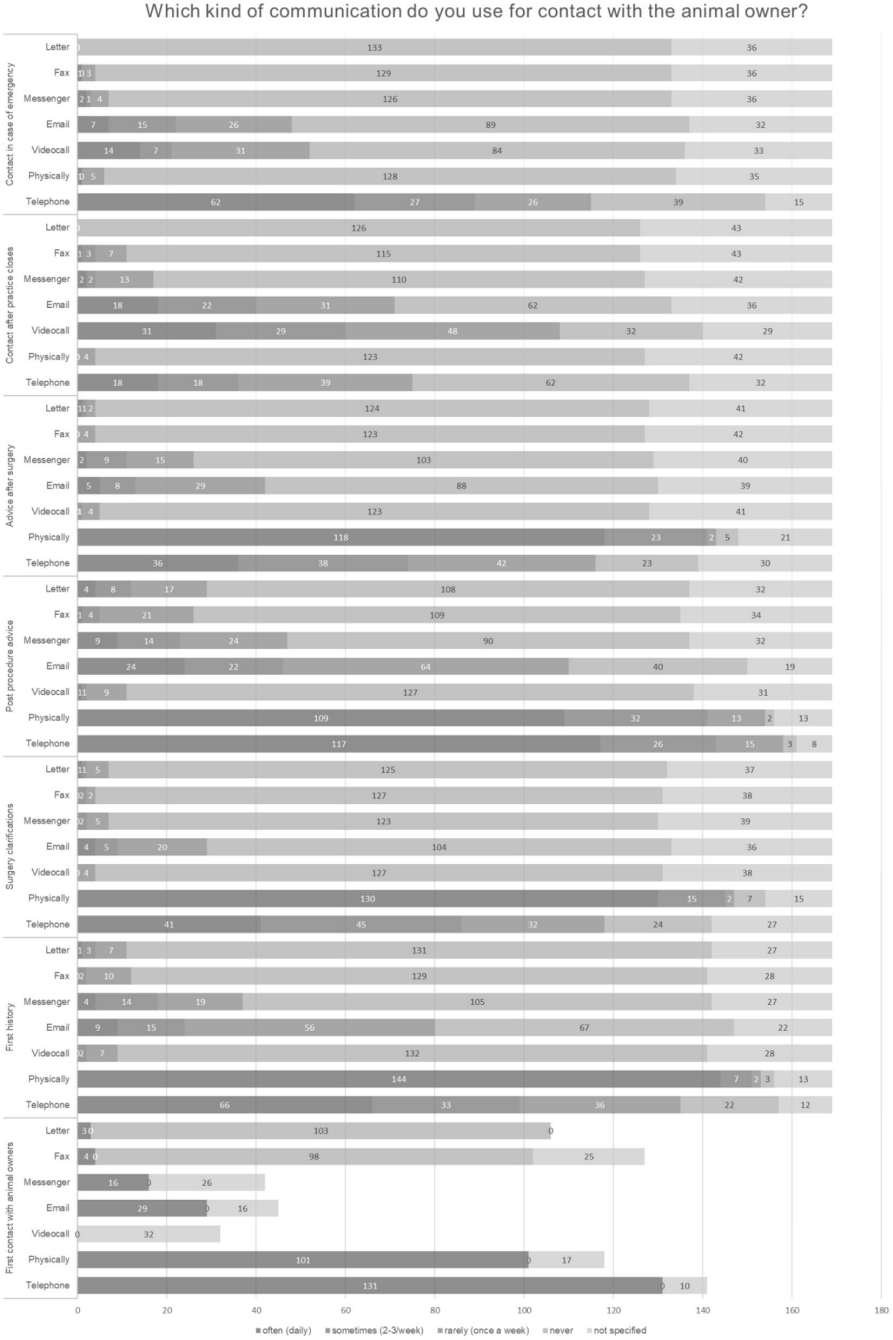
Kind of communication with the owner (*N* = 169).

As shown in Figure 5, face-to-face communication was used in most cases, followed by telephone consultations. E-mails and various forms of messengers were used less frequently. Video telephony was rarely used, fax and letter were not common anymore.

**Figure 6:**
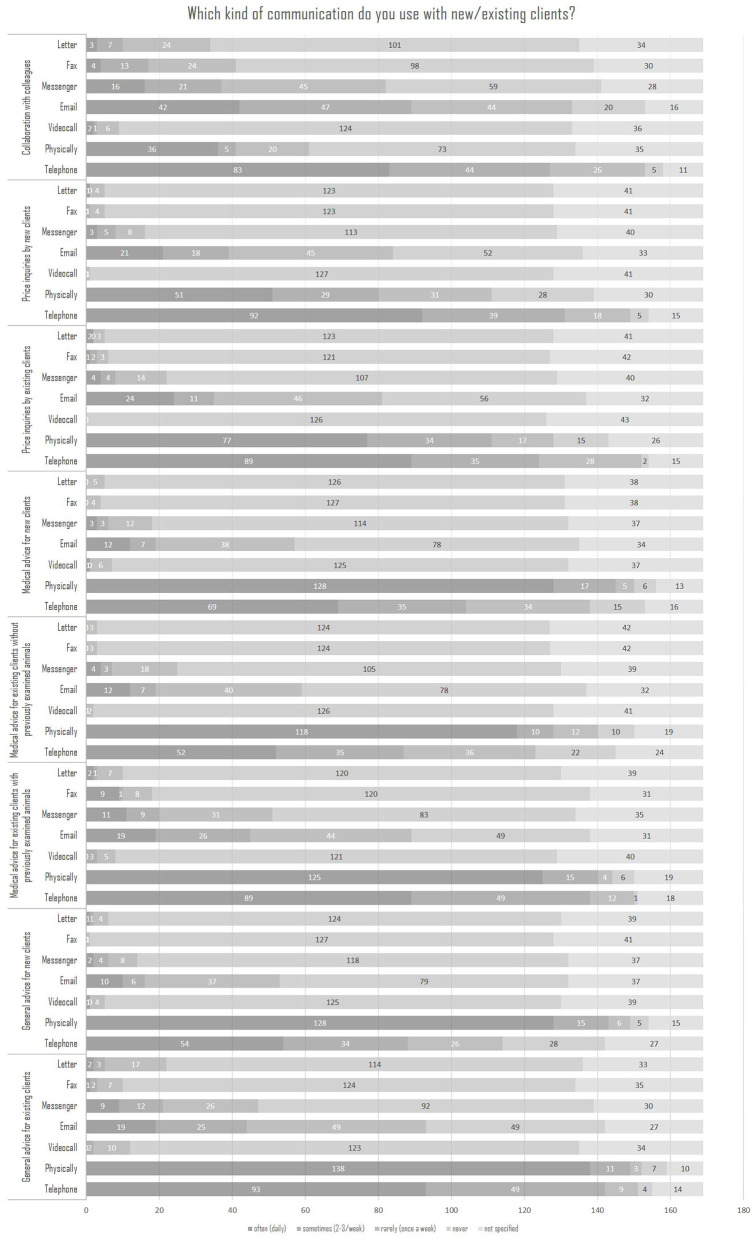
Kind of communication regarding new or existing customers (*N* = 169).

Figure 6 shows again that face-to-face communication was used in most cases, followed by telephone consultations, E-mails and various forms of messengers being used less frequently.

### 3.3. Questions about legal aspects and information by the authorities

**Figure 7:**
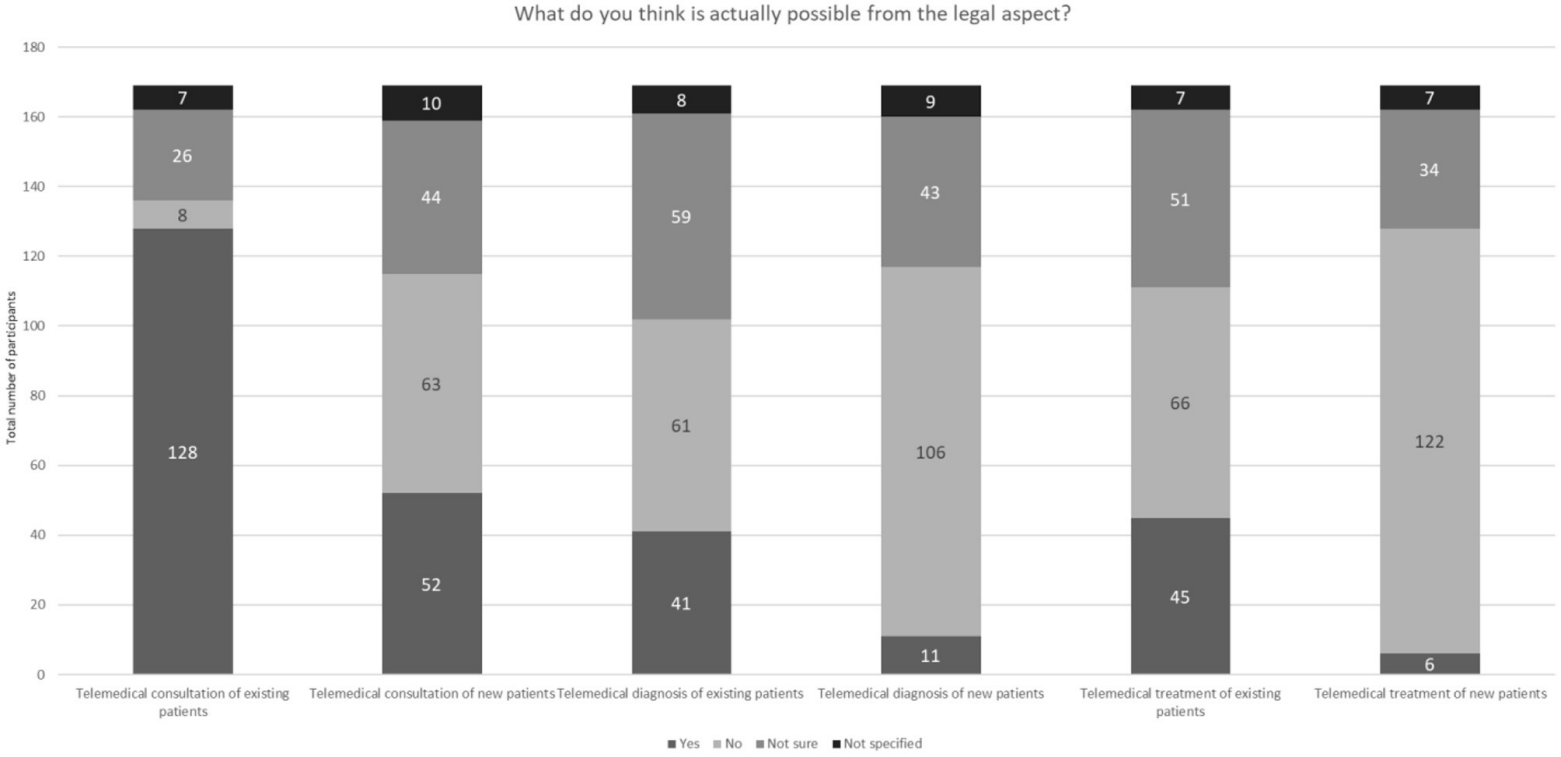
Legal possibilities in veterinary medicine in terms of telemedicine (*N* = 169).

Figure 7 displays the respondents' thoughts about the legally applicable extent of telemedicine. In the analysis, attention was also paid to whether the telemedicine consultation referred to an already existing or to a new doctor-patient relationship.

The majority of study respondents were of the opinion that telemedical care may be used primarily for existing patients. Interestingly, some of the participants thought that both telemedical diagnosis and telemedical treatment of patients were allowed. A significantly large proportion of the respondents were unsure what was legally permitted and which actions were not permitted.

**Figure 8:**
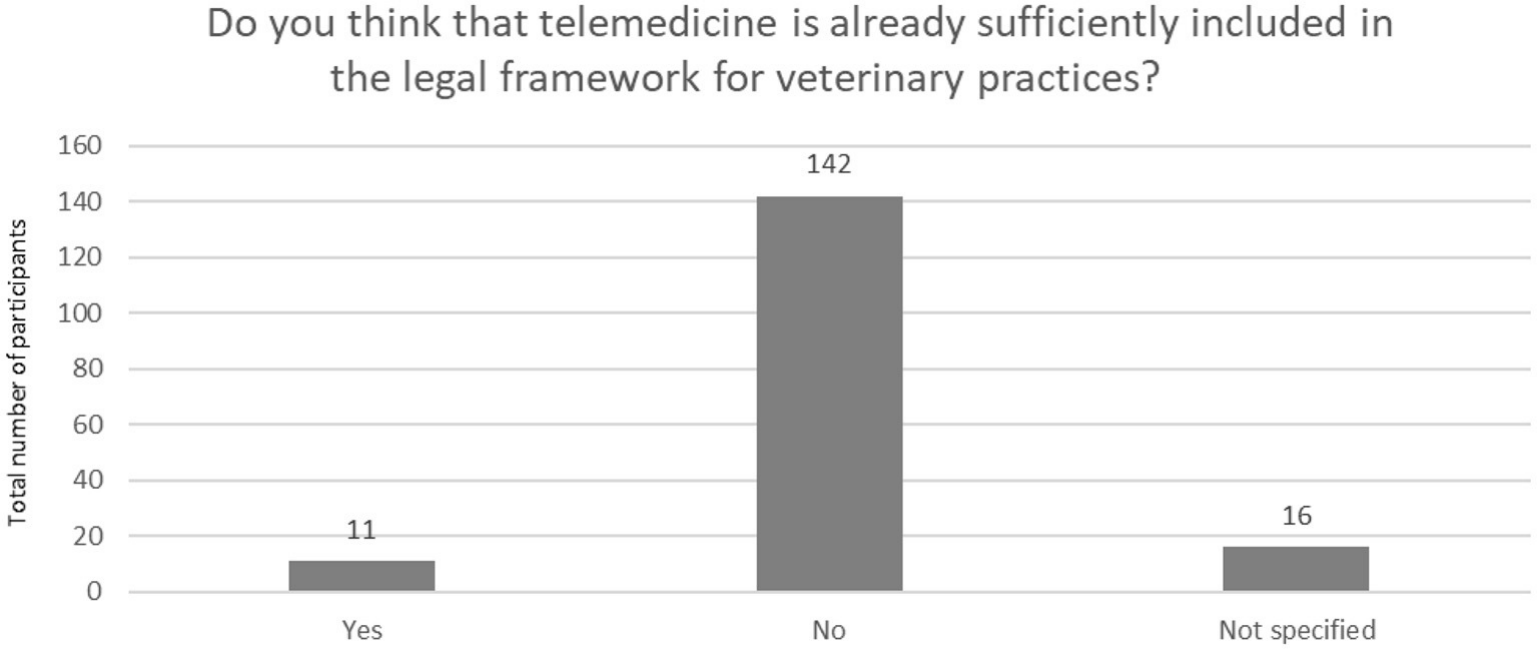
Inclusion of veterinary telemedicine in the legal framework for veterinary practices (*N* = 169).

Figure 8 highlights that only a small percentage of the respondents (*n* = 11, 6.5 %) held the opinion that telemedicine was sufficiently included in the legal framework for veterinary practices, but the majority rated that telemedicine was insufficiently included (*n* = 158, 93.5 %) in the professional regulations.

**Figure 9:**
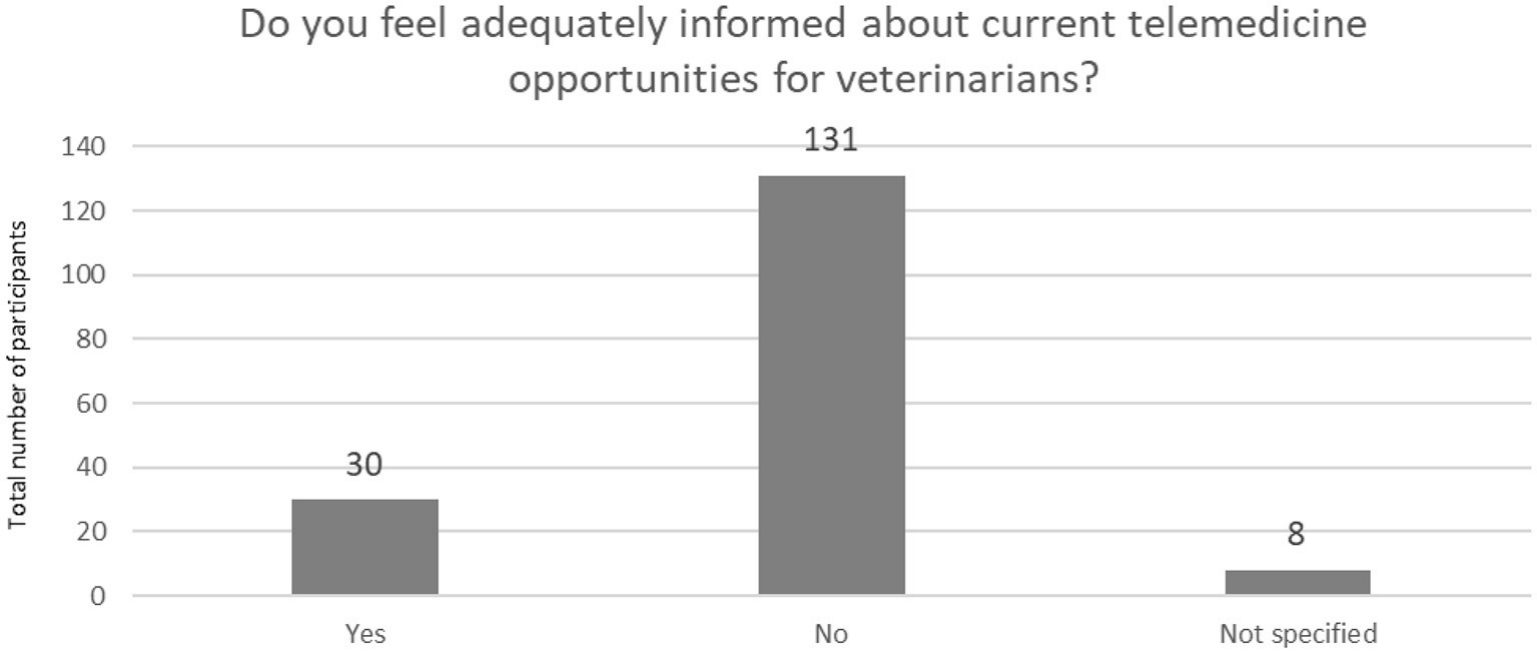
Information about current telemedicine opportunities for veterinarians (*N* = 169).

Also, the majority of respondents (*n* = 131, 77.5 %) do not feel sufficiently informed about current telemedicine opportunities for veterinarians (Figure 9).

**Figure 10:**
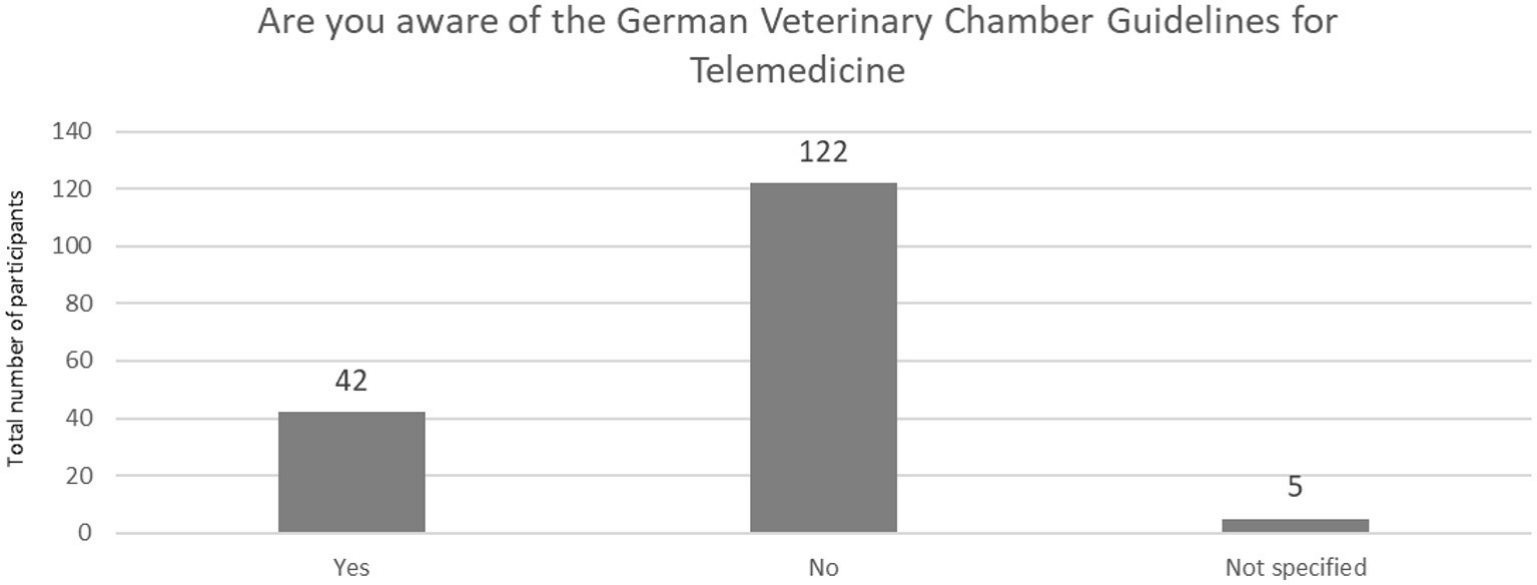
Awareness of the German veterinary chamber guidelines for telemedicine (*N* = 169).

Most participants were not aware of the Guidelines_of_the_Ad-hoc-AG-Telemedizin by the German Veterinary Chamber (1) (*n* = 122, 72.19 %) (Figure 10), which was released just one month before the survey was sent.

**Figure 11:**
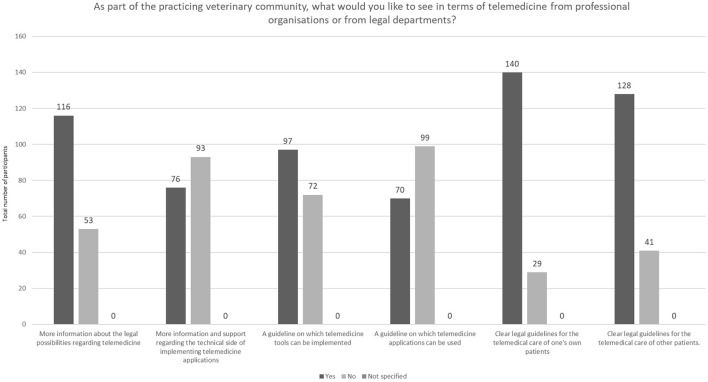
Needed support and information by legal departments (*N* = 169).

In addition to more information about the legally protected possibilities in relation to telemedicine, the majority of participants would also like to see clear remaining guidelines for using telemedicine in practice. Technical assistance and support were less in demand (Figure 11).

### 3.4. Questions related to COVID-19 as an influencing factor

**Figure 12:**
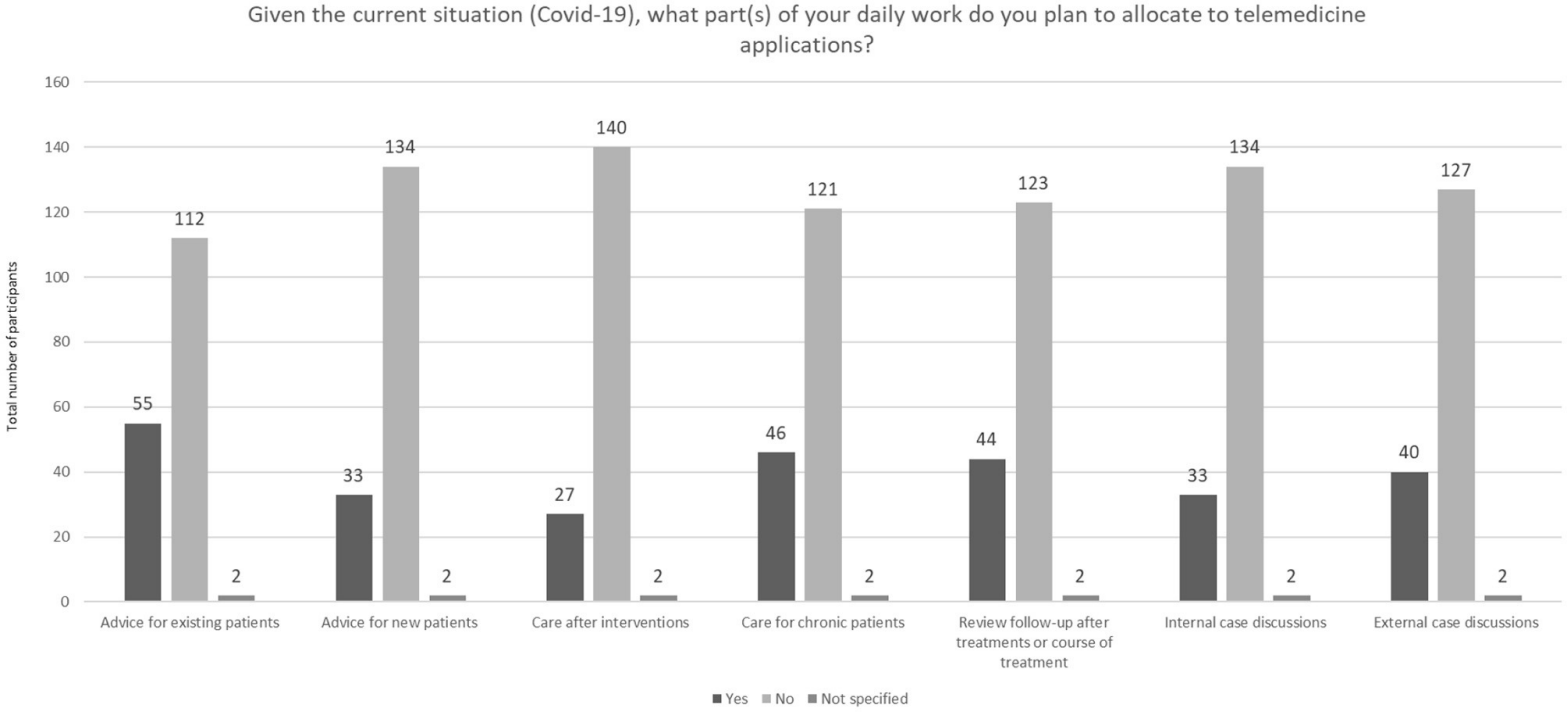
Allocation of daily work procedures to telemedical applications given the current situation (COVID-19) (*N* = 169).

The majority of respondents did not want to or intend to use telemedicine applications during their consultations, control- or follow-up-appointments or case discussions only because of the COVID-19 pandemic (Figure 12).

## 4. Discussion

The current study investigated the usage and awareness of telemedicine and its legal framework among German veterinarians. According to socio-demographic aspects, mostly women who worked in a small animal practice or ran one in a large city were interested in telemedicine in general. It is also interesting that veterinarians who were on parental leave were interested in working with telemedicine. Presumably, these veterinarians want to explore alternative employment models and telemedicine could be at least one of the possibilities.

According to the respondents' answers, digital/telemedical solutions were used already, but did not prominently feature in the daily veterinary medical practice. In contrast, face-to-face and telephone consultations were still the most frequently used communication channels with animal owners.

The only exception was teleradiology, which is well established in the area of doctor-to-doctor communication and has been used for more than 30 years (4, 5). Here, the technical and legal framework seems to be more clearly defined and can presumably be implemented well in existing practice structures due to more uniform technical approaches (5).

With regard to the legal framework, which was also a central component of the survey, transparency and further communication are expected and needed. As other authors have shown in relation to initial developments during the COVID-19 crisis, there is significantly less clarity concerning the legal framework in veterinary medicine than in other fields (21). Legal clarification for the authorization of digital procedures for different veterinary medical situations is still lacking and seems to impose a corresponding constraint on practitioners (30). While preparing the survey, first guidelines were discussed and published by the authorities (1) so that this could be the reason for the low awareness of them, as the survey was conducted close to the publication date.

With regard to the implementation of veterinary telehealth and telemedicine research, further research is needed (21). While detailed analyses are available in human medicine (9, 16), in veterinary medicine the topic seems to be less developed. However, at the same time at least in practice, telemedicine approaches are becoming increasingly important (21). Scientific research in this regard is mainly limited to overview articles or forecasts and therefore do not offer any empirically robust statements about the actual implementation status or acceptance of corresponding solutions (16). Only teleradiology, as a technically oriented solution, is an exception. Nevertheless, especially the implementation of corresponding solutions also depends on legal, social and societal framework conditions (12, 13).

In addition, a recent analysis for identifying and analyzing strengths, weaknesses, opportunities and threats (SWOT analysis) performed by a group of experts from the European Coordinating Committee on Veterinary Training identified the urgency to “develop policies and regulations to ensure efficient, safe, ethic and legal use of digital technologies and artificial intelligence in veterinary medicine” (17).

It remains unclear to what extent these framework conditions are also related to different remits and approaches in terms of location (urban vs. rural) and orientation of practices (large/small animals). For example, it can be argued that veterinary practices in rural areas dominated by livestock farming, sometimes differ significantly from those in urban areas (16). It is therefore clear that the approaches to telemedicine taken by practitioners in these two areas should also differ. Approaches in One Health, animal disease control or zoonoses are also relevant areas for which recommendations and standards should be developed (11).

One limitation of the study may be the empirical consideration of the legal framework. While the literature on telemedicine and veterinary telemedicine states that the legal framework and clarity concerning legal and technical aspects are of fundamental importance for the implementation status, the empirical findings presented here only confirm this to some extent (4, 9). The actual legal framework conditions are not well publicized. Also, a global comparative study is advisable, which not only examines the internal national differences in perception and interpretation of the framework situation, but also the actual differences with regard to legislation, comparing these with the implementation status of telemedicine solutions.

In particular, it is advisable that future research in the field focuses on the perspective of clients. The present study showed the implementation status and the related expectations from the perspective of the practicing veterinarians. For a successful implementation of telemedical solutions, it is relevant to include the perspective of the animal owners, which has corresponding implications especially for the practical work. A deeper understanding of this perspective and the needs of this important group of stakeholders should be taken into account in order to be able to develop telemedical solutions tailored to their wishes.

To establish a telemedical and digital mindset in the veterinarian eco-system, it is recommended to create a clear framework as a guideline for veterinarians.

The guideline may include the following topics:

1. Reference to transparent and well-defined legal basis and definitions with a clear differentiation between teleconsultation, teletreatment and telediagnosis.
2. Recommendation regarding the selection and transformation of previous analog processes in digital or digital/telemedicine-supported processes.
3. Building awareness regarding telemedicine, its possibilities and its advantages.
4. Expanding available guidelines regarding telemedicine.
5. Creating billing regulations for veterinarians.

At the same time, it seems reasonable to formulate additional concrete questions for future surveys, since ignorance about telemedicine, skepticism or unclear understanding *per se* may have an influence on the answers.

In addition to skepticism about digitalized veterinary medicine, the research results also indicate adherence to “old” or analog forms of communication. A recently published study “Digitalized Veterinary Medicine 2030” also shows clear tendencies and results in this regard (27). It states: “Currently, there is a digital divide between practitioners and industry representatives in veterinary medicine. The study results also show that there is a considerable gap in the learning strategies used and positions taken on digitalization within the groups of practitioners studied. Dealing with the opportunities and risks of digitalization in veterinary medicine, however, requires a willingness to learn, curiosity and a willingness to experiment from many more actors. For the future viability of veterinary medicine, a “digital mobilization” of practices and clinics therefore appears to be important.” (27). It continues: “In this context, the animal health industry and a still relatively small number of practitioners with a high level of digital competence in the sector are in a pioneering position. These actors have an important potential to support practices, clinics and teaching and to promote the transfer of digital knowledge. But the strategic and political role of professional associations is also important in terms of targeted development of digital competence.” (27).

This means that currently only a few innovative players with smaller satellite ideas are actively trying to advance digitalization and telemedicine in veterinary medicine and there is a lack of large-scale solutions. Professional associations such as the BTK (German Veterinary Association) or the BPT (German Association of Practicing Veterinarians) have taken the first steps and published a guideline on the use of telemedicine in the veterinary profession. In addition, more digital competence must be taught and the use of telemedical tools must be encouraged in the field of education and training.

In summary, telemedicine comprises only a small part of the veterinary service, namely the provision of medical information when doctors and patients are physically distant and the exchange of medical information occurs across distances. In view of the general digitalization of many processes in medicine, telemedicine should be considered as an essential but not the only part of this development.

Functioning, effective telemedical services can only be used adequately and in a result-oriented manner in a digital and professional environment. Corresponding tools are already available on the market and are constantly being further developed.

Integration of digital technologies can improve many parts of the veterinarian work in all kinds of practices. At the same time, however, the willingness of veterinarians and their clients to use these tools must be promoted (17).

Many new opportunities are already given, such as artificial intelligence and wearable home diagnostic devices. If the framework is clear and easy to adapt, telemedicine is a huge option rather than a threat, as it can establish a state-of-the-art connection between doctors, animal owners and animals to raise the overall health of animals. “Ignoring it would be deleterious for the future of the veterinary profession” (17).

## Data availability statement

The raw data supporting the conclusions of this article will be made available by the authors, without undue reservation.

## Ethics statement

The studies involving human participants were reviewed and approved by Universities Ethics Committee and approved prior to investigation. The participants provided informed consent to participate in this study. This study was conducted according to the ethical standards of the Stiftung Tierärztliche Hochschule Hanover, Germany. The Doctoral Committee of the University, validated the project according to the ethical guidelines for research with human participants and approved the study. The university's data protection officer approved the project. The voluntary participants consented to the processing of their data according to the EU General Data Protection Regulation of 2018 (General Data Protection Regulation Art. 6 I 1 lit. e i.V.m. 89 and Lower Saxony Data Protection Act § 3 I 1 No. 1 NHG, § 13). Data processing was carried out anonymously in accordance with the University's data protection regulations.. Written informed consent for participation was not required for this study in accordance with the national legislation and the institutional requirements. Written informed consent was not obtained from the individual(s) for the publication of any potentially identifiable images or data included in this article.

## Author contributions

BB designed and conducted the study, performed formal analysis, and prepared the original draft manuscript. AT, JE, and CK reviewed the study design. CK supervised the study and validated the analysis. All authors reviewed, edited the manuscript, contributed to the article, and approved the submitted version.

## References

[1] Bundestierärztekammer. Telemedizin. (2020). Available online at: https://www.bundestieraerztekammer.de/Leitlinien_der_Ad-hoc-AG-Telemedizin_-_Stand_28_Jan_2022_0.pdf (accessed January 10, 2022).

[2] Hess L. Telemedicine: the future of veterinary practice. J Avian Med Surg. (2017) 31:165–71. 10.1647/1082-6742-31.2.165 28644088

[3] Papageorges M Tilley L. Why telemedicine? Clin Tech Small Anim Pract. (2001). 16:90–4. 10.1053/svms.2001.26225 11503456

[4] Tilley LP Miller MS Wortman JA Biery DN Coward BH. Teleradiology in veterinary medicine. Vet Radiol Ultrasound. (1986) 1:172–5. 10.1111/j.1740-8261.2007.00331.x 3321283

[5] Poteet BA. Veterinary teleradiology. Vet Radiol Ultrasound. (2008) 49:S33–SS6. 18283984 10.1111/j.1740-8261.2007.00331.x

[6] Clark K. Innovation is key to the future success and sustainability of the veterinary profession. Vet Rec. (2017) 180:236. 10.1136/vr.j1185

[7] Hwang S Song Y Kim J. Evaluation of AI-assisted telemedicine service using a mobile pet application. Appl Sci. (2021) 11:2707. 10.3390/app11062707

[8] Murphy T. Telemedicine's challenge: getting patients to click the app. AP News. (2019) 2:7. Available online at: https://apnews.com/article/ap-top-news-us-news-health-north-america-business-7baf70c5ce2c4f0eb928596aa1ac5e6b (accessed January 09, 2023).

[9] Mills G. Telemedicine survey reveals vets' concerns. Vet Rec. (2018) 182:445. 10.1136/vr.k1725 29674458

[10] Sprague L. Telehealth: into the mainstream? NHPF. (2014) 853:1−15. Available online at: https://hsrc.himmelfarb.gwu.edu/sphhs_centers_nhpf/285/ (accessed January 09, 2023).

[11] Pathak A Kumar D. Telehealth in India: helping to achieve health for all. Vet Rec. (2017) 180:572–3. 10.1136/vr.j2219 28600418

[12] Martinelli M. Digital imaging advances and the future. Vet Clin North Am Equine Pract. (2001) 17:275–95. 10.1016/S0749-0739(17)30062-7 15658176

[13] Ullrich L. Radiologie auf modernen (Ab)wegen [Radiology (gone astray) on modern paths]. Der Radiologe. (2021) 61:1028–30. 10.1007/s00117-021-00914-3 34613442 PMC8493777

[14] Spieren S. Jetzt kommt es auf uns Hausärzte an! Coronavirus in Deutschland. MMW Fortschritte der Medizin. (2020) 162:41. 10.1007/s15006-020-0338-6 32248473 PMC7127841

[15] Devi S Singh RD Ghasura RS Sharma MK Sharma MC. Telemedicine: a new rise of hope to animal health care sector - A Review. Agricultural Reviews (2015). 36(2). p. 153–158. 10.5958/0976-0741.2015.00018.5

[16] Gyles C. Veterinary telemedicine. Can Vet J. (2019) 60:119. 30705446 PMC6340261

[17] ECCVT Report. European Coordinating Committee on Veterinary Training. Report of the ECCVT expert working group on the impact of digital technologies & artificial intelligence in veterinary education and practice. Available online at: http://www.veterinary.ankara.edu.tr/wp-content/uploads/sites/593/2020/10/EK-4.pdf (accessed January 10, 2022).

[18] Iivari N Sharma S Ventä-Olkkonen L. Digital transformation of everyday life–How COVID-19 pandemic transformed the basic education of the young generation and why information management research should care? Int J Inf Manage. (2020) 55:102183. 10.1016/j.ijinfomgt.2020.102183 32836640 PMC7320701

[19] Nagel L. The influence of the COVID-19 pandemic on the digital transformation of work. Int J Sociol Soc Policy. (2020) 40:861–75. 10.1108/IJSSP-07-2020-0323

[20] Wagman BA. Animal law, shelters, and veterinary care in the time of the virus. In: Animal Law. Portland, OR: Joe Christensen, Inc. (2020). p. 13.

[21] Teller LM Moberly HK. Veterinary telemedicine: a literature review. Vet Evid. (2020). 5:1–26. 10.18849/ve.v5i4.349

[22] Scott Kruse C Karem P Shifflett K Vegi L Ravi K Brooks M. Evaluating barriers to adopting telemedicine worldwide: a systematic review. Journal of telemedicine and telecare (2018) 24:4–12. 10.1177/1357633X16674087 29320966 PMC5768250

[23] Mars M Auer REJ. Telemedicine in veterinary practice. J S Afr Vet Assoc. (2006) 77:75–8. 10.4102/jsava.v77i2.348 17120623

[24] Schur LA Ameri M Kruse D. Telework after COVID-19: a “silver lining” for workers with disabilities? J Occup Rehabil. (2020) 30:521–36. 10.1007/s10926-020-09936-5 33156435 PMC7645902

[25] Okubo T. Spread of COVID-19 and telework: evidence from Japan. COVID-19 Econo. (2020) 32:1–25.

[26] Australasian for HIV,. Viral Hepatitis Sexual Health Medicine, Telehealth Practice Online Survey. (2020) Available online at: https://www.surveymonkey.com/r/COVID-19Telehealth (accessed October 1, 2021).

[27] Dessauer Zukunftskreis,. Studie Digitalisierte Veterinärmedizin Status Quo. (2020). Available online at: https://www.dessauer-zukunftskreis.de/fileadmin/zukunftskreis_2016/files_open/Studie_Digitalisierte_Vetmed_2020_TK_JH.pdf (accessed December 15, 2020).

[28] DAK-Gesundheit. Ärzte Zeitung. Digitalisierung im Gesundheitswesen: Skepsis von Ärztinnen und Ärzten überwinden. Available online at: https://www.dak.de/dak/bundesthemen/digitalisierung-im-gesundheitswesen-skepsis-von-aerztinnen-und-aerzten-ueberwinden-2524736.html#/ (accessed April 15, 2020).

[29] Lenzner T Neuert C Otto W. Kognitives Pretesting. Mannheim, GESIS – Leibniz-Institut für Sozialwissenschaften (SDM Survey Guidelines). GESIS-Leibnitz Institute for the Social Sciences, Mannheim, Germany (2015).

[30] Hawk C. Veterinary Telemedicine Perception and Utilization Intention. University of Tennessee Honors Thesis Projects (2018). Available online at: https://trace.tennessee.edu/utk_chanhonoproj/ 2234 (accessed October, 2021).

